# Barriers to Education for Children With Neurodisabilities in a Developing Country

**DOI:** 10.7759/cureus.54693

**Published:** 2024-02-22

**Authors:** Kai Lin Gan, Teik Beng Khoo

**Affiliations:** 1 Paediatric Neurology Unit, Hospital Tunku Azizah, Kuala Lumpur, MYS

**Keywords:** barriers, education, school, children, neurodisability

## Abstract

Despite having equal rights to education, children with neurodisability (CWND) continue to face a myriad of barriers to education. This cross-sectional survey aimed to investigate the barriers to education among CWND who attended follow-up at a Malaysian tertiary hospital. Sixty-two caregivers of CWND completed a questionnaire which included the ‘Washington Group/United Nations Children's Fund (UNICEF) Child Functioning Module’, type of schooling and open-ended questions for barriers to education. The out-of-school rate was 16/62 (26%). The level of schooling (i.e. not in school, special education or mainstream class) was strongly associated with difficulties in mobility, self-care, communication, controlling behaviour and getting along with other children. Five themes identified by caregivers as barriers to education were ‘school infrastructure’, ‘inclusive opportunity, staffing and teacher’s training’, ‘social environment’, ‘child’s intrinsic factor’ and ‘practical limitations’. Practical solutions to overcome the significant barriers to education are required to address the unmet educational needs of CWND.

## Introduction

Neurodisability refers to a group of congenital or acquired chronic neurological conditions originating either from the brain and/or neuromuscular system which can lead to functional difficulties [1]. It includes conditions such as cerebral palsy, epilepsy, acquired brain injury, intellectual disability, autism spectrum disorder and attention deficit hyperactive disorder [1].

In Malaysia, persons with disabilities can be registered with the Department of Social Welfare under seven categories: visual, hearing, speech, physical, learning, mental and multiple disabilities. By 31 January 2023, there were 152,065 children and young people under the age of 19 years registered with the Department of Social Welfare as ‘persons with disability’ [2]. Among this group of children and young people, 14,195 (9.3%) were registered in the 'physical' category and 117,900 (77.5%) in the 'learning disabilities' category [2]. This would suggest an estimated prevalence of 1.5 per 1,000 population and 12.9 per 1,000 in these two categories, against a total population of about 9.13 million Malaysian children [3]. Given that neurodisability can result in both physical impairment and learning disability, the largest group of children with a disability may well consist of children with neurodisability. These figures may even be underestimated due to a lack of population-level data [4]. In a population-based survey by Khoo et al. [5], the prevalence of physical disability among Malaysian children aged between seven and 17 years was 2.8 per 1,000 population.

Disability and functioning are influenced by the interaction between health, personal and environmental factors [6]. The International Classification of Functioning, Disability and Health (ICF) is a comprehensive framework developed by the World Health Organisation in 2001 to classify an individual’s functioning and disability levels in the form of bodily function, participation and activity, utilising a holistic bio-psycho-social model [6]. Based on this framework, disability becomes apparent when a person with functional limitations is faced with environmental barriers that hinder their participation, compared to others without functional limitations. To enable better measurement of child functioning, the United Nations Children’s Fund (UNICEF) and the Washington Group on Disability Statistics (WG) developed the ‘Washington Group/UNICEF Child Functioning Module’ (CFM) in 2016 which conforms to the ICF framework [7]. Instead of focusing on medical conditions or body function, it consists of a series of questions aiming to evaluate various forms of functional difficulties encountered by children between the ages of two and 17 years. This is because children with the same diagnosis may experience different functional limitations [7]. These domains include hearing, vision, communication or comprehension, learning, mobility and emotions. The purpose of CFM is to help identify those who are prone to reduced participation in non-accommodating environments and it can be used in household or institutional research to facilitate policy design and implementation [7]. Utilising this module, a community-based prevalence study done by the Malaysian Institute for Public Health, ‘The National Health and Morbidity Survey (NHMS) 2019’, reported that 4.7% of children in Malaysia aged between two and 17 years experienced functional difficulty in at least one of the domains assessed within the module [8]. This figure is similar to the disability prevalence reported in a UNICEF study which estimated a prevalence of at least 5.5% of children younger than 18 years in Europe and Central Asia living with disability [9].

For children with neurodisability, a myriad of factors including physical impairment, communication difficulties, cognitive deficit and behavioural regulation issues can impact on home, societal and educational participation [1]. Education in particular is pivotal to enable the acquisition of academic knowledge and life skills among this group of children which can bring positive effects on long-term health outcomes as well as catalysing social and economic participation in the future [10]. Article 24 of the Convention on the Rights of Persons with Disabilities (CRPD) of the United Nations stipulates that 'States Parties recognise the rights of persons with disabilities to education … without discrimination and on the basis of equal opportunity' and 'ensure an inclusive education system at all levels and lifelong learning'. Yet a study of 30 developing countries found that children with disabilities are 10 times less likely to attend school than children without disabilities [11]. In Malaysia, the study by Khoo et al. found that about 31.5% of Malaysian children with physical disability did not receive formal education as compared to 1.7% of children without physical disability [5]. 'Children Out of School' report which is based on a study done as part of the global Out-of-School Children Initiative (OOSCI) by UNICEF and United Nations Educational, Scientific and Cultural Organisation (UNESCO) stated that between 50.1% and 69.3% of registered school-aged children with disabilities in the state of Sabah, Malaysia, were more likely to be absent from school compared to between 9% and 16.7% among children without disabilities [12]. For those who attend school, many face significant barriers to education [10]. Previous studies identified not only personal and health factors, but also environmental determinants such as inadequate teacher training, infrastructural barriers, financial difficulties, attitudinal factors and school bullying [10,13].

The present study

Although there had been previous studies looking at schooling rates and barriers to education among children with disability, there is currently a lack of local data specific to children with neurodisability who are expected to have higher levels of functional difficulties compared to the general population. The present study aims to look at the levels of disability and functioning among children with neurodisability using CFM. This study also aims to explore the association of CFM with the level of schooling, with barriers and challenges that these children encountered in school and the reasons underpinning non-schooling among this group of children. The data from this exploratory study could facilitate a more definitive, larger sample or population-level study in the future. Ultimately, it is hoped that findings from this study could be useful for service providers of children with neurodisability including healthcare professionals, educators, welfare officers and policy-makers to enable the delivery of a more barrier-free schooling experience for this group of children.

## Materials and methods

Procedures

This is a cross-sectional survey study among caregivers of children with neurodisability (CWND) attending the outpatient clinics of Hospital Tunku Azizah, Kuala Lumpur. CWND included any children with congenital or acquired chronic neurological conditions originating either from the brain and/or neuromuscular system such as cerebral palsy, neuromuscular disease, epilepsy, acquired brain injury, intellectual disability and autism spectrum disorder. CWND aged between five and 17 years old attending the outpatient clinics (including general paediatric neurology clinics, cerebral palsy clinics, muscle clinics, tuberous sclerosis clinics, spina bifida clinics and child development clinics) were eligible for this study. Caregivers of CWND who were identified as potential participants are invited to take part in the study during clinic visits. While waiting for the clinic appointment, they were provided with an information sheet about the study to read and given the opportunity to ask questions. If they agreed to participate, written informed consent from caregivers was sought by the principal investigator in a face-to-face encounter. Following consent, caregivers completed a one-off paper-based questionnaire in Malay language consisting of closed and open-ended questions administered by the principal investigator, who was present to assist with the completion of the questionnaire if required (for example, if the caregiver did not understand a particular question). The English equivalent of the questionnaire is available at this link: https://data.unicef.org/wp-content/uploads/2022/05/Child_Functioning_for_Children_Age_5_to-17_May-2022_FINAL.docx. The estimated duration to complete the questionnaire was around 15 minutes, after which participants are seen in the clinic they were attending. The approval for this study was granted by the Medical Research & Ethics Committee, Malaysia Ministry of Health (reference no: NMRR-20-2137-56612).

Participants

Caregivers were approached during outpatient clinics (including general paediatric neurology clinics, cerebral palsy clinics, muscle clinics, tuberous sclerosis clinics, spina bifida clinics and child development clinics) in Hospital Tunku Azizah in Kuala Lumpur, Malaysia, between November 2020 and June 2021. The target sample size was 60 surveys from caregivers. There is no consensus regarding the sample size in questionnaire studies, but the general rule of thumb is to use at least 30 subjects to estimate a parameter in a pilot study [14]. Therefore, a sample size of 60 was thought to be pragmatic and could be realistically recruited during the proposed study period within a single study site.

Measures

Demographics of the Child and Caregiver

The child’s diagnosis and age were collected from the hospital database. The questionnaire in this study collected data on caregivers including parental status i.e. father or mother, age, education level, marital status and household income, and the place of residence. 

Schooling Details, Participation and Experience

Caregivers were also asked to provide details of the type of school that the child was currently enrolled in. For children not attending school, an open-ended question was used to capture the reasons for being out of school. For children in school, four yes/no questions were used to capture information on the child’s schooling experience, including whether the child was regularly attending school (with >90% average attendance), the child felt accepted at school, the child was interested in attending school and the occurrence of bullying at school. To capture information on school participation, caregivers were asked to rate their child as either ‘same as the child’s classmates’ or ‘more difficult compared to the child’s classmate’ in six aspects including academic activities with classmates, play and social activities with classmates, movement around the school compound, usage of learning materials, communications with other children in school and communications with adults in school.

Child Functioning

Child functioning was measured with the ‘Washington group/UNICEF Module on Child Functioning’, which evaluates 12 domains with 24 items. The domains are divided into ‘basic functions’ which has seven domains evaluated with 18 items (self-care, learning, remembering, three items for hearing, three items for vision, two items for communication, and seven items for mobility) and ‘complex function’ which has five domains evaluated with six items (controlling behaviours, attention and concentration, coping with change, relationships and two items for affect, i.e. anxiety and depression). All items were rated by the caregiver on a four-point scale (‘no difficulty’, ‘some difficulty’, ‘a lot of difficulty’ and ‘cannot do’) except for the two items on affect which were rated on a five-point scale according to the frequency of anxiety or depression (‘daily’, ‘weekly’, ‘monthly’, ‘a few times a year’ and ‘never’). The version of the Washington group/UNICEF Module on Child Functioning used was the Malay language version, translated from English by the National Institutes of Health (NIH) Ministry of Health Malaysia for the National Health and Morbidity Survey 2019 [8].

Barriers and Facilitators

Open-ended questions regarding barriers encountered in school and facilitators which would help with a child’s schooling were included in the questionnaire. The two questions were adequately open to capture new insights: ‘please state the barriers faced by your child in school’ and ‘please state the facilitating factors which can help your child in school to overcome his or her challenges’.

Analysis

The data analysis was done using the SPSS version 25 (IBM Corp, Armonk, NY, USA). Descriptive data are expressed as median (interquartile range, IQR) for continuous variables and as proportion for categorical data. The Washington group/UNICEF Module on Child Functioning questionnaire were analysed according to the recommendations of UNICEF data (https://data.unicef.org/resources/module-child-functioning-tabulation-plan-narrative/), and the results for each domain were tabulated.

Since only a small proportion of the cohort has the most severe level of difficulty, the Washington group/UNICEF Module on Child Functioning were dichotomised into ‘no difficulty’ versus ‘at least some difficulty’ to evaluate the association between each domain of functional difficulties and the level of schooling (not in school, special education and mainstream class). Appropriate descriptive statistics were generated for demographics, schooling experience and the levels of disability, including tabulation of demographic data according to the level of schooling. Due to the limited sample size, the Chi-squared test of association was only performed for demographic factors according to the level of schooling where there was an obvious association. A value of p < 0.05 was considered statistically significant.

The responses to the open-ended questions were thematically analysed according to Braun and Clarke’s procedures [15]. The barrier themes were first identified. The principal investigator read the open-ended responses from caregivers several times for familiarisation and generated initial codes with an inductive approach. An analytic framework was developed based on the emerging codes, then pertinent data were extracted using the coding framework. The codes were then organised into broader themes. The principal investigator searched for and reviewed themes (constant comparison to check each theme against the rest of the data). The final form of themes was then constructed and reported. Following the identification of the barriers, facilitators to education were similarly analysed and, where possible, matched to each barrier theme. Of note, any obvious pattern emerging from the initial quantitative analysis according to the levels of disability and schooling would prompt consultation of open-ended response to aid interpretation.

## Results

A total of 62 caregivers of CWND participated in the survey. The median age of the CWND was 9.5 years, and cerebral palsy was the most common diagnosis (N=25, 40.3%). Caregivers with higher levels of education and higher income were more likely to have children in mainstream schools (Table 1).

**Table 1:** Primary diagnosis, demographics and type of school ^1^Chi-square test of association=0.047. ^2^All six single parents were mothers. ^3^Data are missing for six fathers since there are six single parents, all of whom were mothers. ^4^Chi-square test of association=0.316. ^5^Chi-square test of association=0.008. ​​​​​​​^6^Chi-square test of association=0.125. IQR: interquartile range.

Demographics	Not in school (N=16)	Non-mainstream school (N=22)	Mainstream school (N=24)	Overall cohort (N=62)
Age of child in years, median (IQR)	9.5 (6.7-12.8)	9.6 (8.5-13.0)	8.0 (6.8-10.7)	9.5 (7.2-11.8)
Child in schooling age, i.e. ≥7 years, N (%)	12 (75.0)	19 (86.4)	18 (75.0)	49 (79.0)
Female child,^1^ N (%)	8 (50.0)	11 (50.0)	5 (20.8)	24 (38.7)
Primary diagnosis of the child, N (%)				
Cerebral palsy	8 (50.0)	10 (45.5)	7 (29.2)	25 (40.3)
Duchenne muscular dystrophy	3 (18.8)	1 (4.5)	5 (20.8)	9 (14.5)
Tuberous sclerosis	1 (6.3)	3 (13.6)	1 (4.2)	5 (8.1)
Spinal muscular atrophy	0	2 (9.1)	0	2 (3.2)
Ullrich muscular dystrophy	0	0	1 (4.2)	1 (1.6)
Congenital myopathy	0	0	2 (8.3)	2 (3.2)
Non-cerebral palsy hemiparesis	2 (12.5)	2 (9.1)	2 (8.3)	6 (9.7)
Inborn error of metabolism	1 (6.3)	0	3 (12.5)	4 (6.5)
Epilepsy	1 (6.3)	2 (9.1)	1 (4.2)	4 (6.5)
Microphthalmia	0	1 (4.5)	0	1 (1.6)
Global developmental delay	0	0	2 (8.3)	2 (3.2)
Autism	0	1 (4.5)	0	1 (1.6)
Questionnaire completed by mother, N (%)	12 (75.0)	17 (77.3)	19 (79.2)	48 (77.4)
Age of parent in years, median (IQR)	41 (35-46)	40 (37-44)	40 (36-44)	40 (36-44)
Parents are married,^2^ N (%)	15 (93.8)	21 (95.5)	20 (83.3)	56 (90.3)
Education level of the father,^3,4^ N (%)				
Primary school	1 (6.7)	0	0	1 (1.8)
Secondary school	9 (60.0)	15 (71.4)	10 (50.0)	34 (60.7)
Diploma	2 (13.3)	5 (23.8)	6 (30.0)	13 (23.2)
University degree (or above)	3 (20.0)	1 (4.8)	4 (20.0)	8 (14.3)
Education level of the mother,^5^ N (%)				
Primary school	1 (6.3)	1 (4.5)	0	2 (3.2)
Secondary school	11 (68.8)	9 (40.9)	6 (25.0)	26 (41.9)
Diploma	1 (6.3)	7 (31.8)	9 (37.5)	17 (27.4)
University degree (or above)	3 (18.8)	5 (22.7)	9 (37.5)	17 (27.4)
Monthly household income,^6^ N (%)				
Less than RM3,000	6 (37.5)	10 (45.5)	7 (29.2)	23 (37.1)
RM3,000 to RM6,000	8 (50.0)	5 (22.7)	7 (29.2)	20 (32.3)
RM6,001 to RM10,000	1 (6.3)	7 (31.8)	7 (29.2)	15 (24.2)
More than RM10,000	1 (6.3)	0	3 (12.5)	4 (6.5)

Only 24 (38.7%) CWND were in mainstream schools. The majority of the CWND attending schools (N=46) were interested in attending school (N=42, 91.3%), felt accepted at school (N=43, 93.5%) and had similar levels of communication with other children as their peers (N=38, 82.6%). However, only a minority had similar academic (N=20, 43.5%) and social activities (N=20, 43.5%) as their peers (Table 2).

**Table 2:** Detailed types of school attended by participants and schooling experience ^1^No missing data among the 46 children who were attending school. The denominator for the percentage is based on the 46 children who were attending school. ^2^Data are missing for one of the 46 children who were attending school. The denominator for the percentage is based on the 46 children who were attending school.

Type of schools and schooling experience	Overall cohort (N = 62)
Type of school, N (%)	
Not in school	16 (25.8)
Non-mainstream school	22 (35.5)
Early intervention programme (EIP)	2 (3.2)
Community-based rehabilitation (CBR)	3 (4.8)
Special school for the visually impaired	1 (1.6)
Integrated special education programme (primary)	13 (21.0)
Integrated special education programme (secondary)	3 (4.8)
Mainstream school	24 (38.7)
Playschool	1 (1.6)
Mainstream kindergarten	5 (8.1)
Private school	2 (3.2)
Mainstream primary school	15 (24.2)
Mainstream secondary school	1 (1.6)
The child is interested in attending school,^1^ N (%)	42/46 (91.3)
There is regular school absence (<90% average attendance),^1^ N (%)	7/46 (15.2)
The child feels accepted at school,^2^ N (%)	43/46 (93.5)
The child is bullied at school,^2^ N (%)	8/46 (17.4)
The child has similar academic activities as their peers,^2^ N (%)	20/46 (43.5)
The child has similar social activities as their peers,^2^ N (%)	20/46 (43.5)
The child has similar movement within the school as their peers,^2^ N (%)	21/46 (45.7)
The child has similar use of school equipment as their peers,^2^ N (%)	41/46 (89.1)
The child has similar communication with other children as their peers,^2^ N (%)	38/46 (82.6)
The child has similar communication with adults as their peers,^2^ N (%)	36/46 (78.3)

Most (N=36, 58.1%) of the CWND were unable to walk. Only 12 (19.4%) had no difficulty walking (Table 3).

**Table 3:** The level of disability among participants ^1^For this domain, there was a participant with missing data. ^2^Each domain was measured using a four-point ordinal scale ranging from 1 (no difficulty) to 4 (unable to perform/cannot do), except ‘feeling worried/sad’, which was measured using a five-point ordinal scale. ^3^Unlike other domains which were measured using a four-point ordinal scale, ‘Feeling worried/sad’ was measured in a five-point ordinal scale ranging from 1 (‘never experienced’) to 5 (‘every day’). IQR: interquartile range.

Type of disability	Median^2^ (IQR)	No difficulty, N (%)	Some difficulty, N (%)	A lot of difficulty, N (%)	Cannot do, N (%)	
Basic function domains						
Seeing	1 (1-2)	46 (74.2)	11 (17.7)	4 (6.5)	1 (1.6)	
Hearing	1 (1-1)	54 (87.1)	6 (9.7)	0	2 (3.2)	
Walking	4 (2-4)	12 (19.4)	10 (16.1)	4 (6.5)	36 (58.1)	
Self-care^1^	2 (1-3)	19 (31.1)	22 (36.1)	10 (16.4)	10 (16.4)	
Communication	1 (1-3)	34 (54.8)	9 (14.5)	6 (9.7)	13 (21.0)	
Learning	2 (1-3)	24 (38.7)	12 (19.4)	19 (30.6)	7 (11.3)	
Remembering	2 (1-3)	30 (48.4)	12 (19.4)	13 (21.0)	7 (11.3)	
Complex function, emotion and participation domains						
Controlling behaviour	1 (1-2)	37 (59.7)	19 (30.6)	5 (8.1)	1 (1.6)	
Completing a task^1^	1 (1-2)	31 (50.8)	19 (31.1)	7 (11.5)	4 (6.6)	
Accepting change	1 (1-2)	41 (66.1)	16 (25.8)	3 (4.8)	2 (3.2)	
Getting along with other children	1 (1-3)	36 (58.1)	10 (16.1)	6 (9.7)	10 (16.1)	
		Never experienced	A few times a year	Every month	Every week	Every day
Feeling worried/sad^3^	1 (1-2)	32 (51.6)	21 (33.9)	5 (8.1)	3 (4.8)	1 (1.6)

The ability to walk was strongly associated with the level of schooling (p=0.028) (Table 4). Children without difficulty walking were more likely to be in mainstream schools (7/12, 58.3% vs. 17/50, 34%), whereas children with at least some difficulties were much more likely not to be in school (16/50, 32% vs. 0/12).

**Table 4:** The relationship between the type of disability and the type of school attended by the participants ^1^The level of disability is dichotomised into ‘no difficulty’ and ‘at least some difficulty’ because only a small proportion (<20%) of the cohort has the most severe level of difficulty (‘cannot do’) for each type of disability. The only exceptions were 13 participants (21.0%) unable to communicate and 36 participants (58.1%) unable to walk. ^2^For this domain, there was a participant with missing data. ​​​​​​​^3^Unlike other domains which were measured using a Likert scale according to severity (from ‘no difficulty to ‘unable to do‘, ‘Feeling worried/sad’ was measured using a Likert scale according to the frequency (from ‘never experienced’ to ‘every day’).

Type of disability^1^	Not in school, N (%)	Non-mainstream school, N (%)	Main-stream school, N (%)	Chi-squared test of association p-value
Seeing				0.066
No difficulty	11 (23.9)	13 (28.3)	22 (47.8)	
At least some difficulty	5 (31.3)	9 (56,3)	2 (12.5)	
Hearing				0.056
No difficulty	12 (22.2)	19 (35.2)	23 (42.6)	
At least some difficulty	4 (50.0)	3 (37.5)	1 (12.5)	
Walking				0.028
No difficulty	0	5 (41.7)	7 (58,3)	
At least some difficulty	16 (32.0)	17 (34.0)	17 (34.0)	
Self-care^2^				<0.001
No difficulty	0	6 (31.6)	13 (68.4)	
At least some difficulty	15 (35.7)	16 (38.1)	11 (26.2)	
Communication				0.002
No difficulty	5 (14.7)	10 (29.4)	19 (55.9)	
At least some difficulty	11 (39.3)	12 (42.9)	5 (17.9)	
Learning				0.024
No difficulty	4 (16.7)	6 (25.0)	14 (58.3)	
At least some difficulty	12 (31.6)	16 (42.1)	10 (26.3)	
Remembering				0.051
No difficulty	5 (16.7)	10 (33.3)	15 (50.0)	
At least some difficulty	11 (34.4)	12 (37.5)	9 (28.1)	
Controlling behaviour				0.008
No difficulty	6 (16.2)	12 (32.4)	19 (51.4)	
At least some difficulty	10 (40.0)	10 (40.0)	5 (20.0)	
Completing a task^2^				0.016
No difficulty	5 (16.1)	9 (29.0)	17 (54.8)	
At least some difficulty	10 (33.3)	13 (43.3)	7 (23.3)	
Accepting change				0.812
No difficulty	12 (29.3)	11 (26.8)	18 (43.9)	
At least some difficulty	4 (19.0)	11 (52.4)	6 (28.6)	
Getting along with other children				<0.001
No difficulty	4 (11.1)	11 (30.6)	21 (58.3)	
At least some difficulty	12 (46.2)	11 (42.3)	3 (11.5)	
Feeling worried/sad^3^				0.782
Never experienced	9 (28.1)	9 (28.1)	14 (43.8)	
At least a few times a year	7 (23.3)	13 (43.3)	10 (33.3)	

Similar findings were present among 49 CWND aged seven years or older (Table 5): 6/11 (54.5% without difficulty walking were in mainstream schools (vs. 12/38, 31.6%), while 12/38 (31.6%) with at least some difficulties were not in school (vs. 0/11). The out-of-school rate among CWND aged seven or more was 12/49 (24.5%). The majority of them were wheelchair users (10/12, 83%).

**Table 5:** The relationship between the type of disability and the type of school attended by participants aged ≥7 years ^1^The level of disability is dichotomised into ‘no difficulty’ and ‘at least some difficulty’ because only a small proportion (<20%) of the children aged ≥7 years has the most severe level of difficulty (‘cannot do’) for each type of disability. The only exception was 27 participants (55.1%) unable to walk. ^2^For this domain, there was a participant with missing data. ^3^Unlike other domains which were measured using a Likert scale according to severity (from ‘no difficulty to ‘unable to do‘, ‘Feeling worried/sad’ was measured using a Likert scale according to frequency (from ‘never experienced’ to ‘every day’).

Type of disability^1^	Not in school, N (%)	Non-mainstream school, N (%)	Mainstream school, N (%)	Chi-squared test of association p-value
Seeing				0.143
No difficulty	9 (23.1)	13 (33.3)	17 (43.6)	
At least some difficulty	3 (30.0)	6 (60.0)	1 (10.0)	
Hearing				0.333
No difficulty	10 (23.3)	16 (37.2)	17 (39.5)	
At least some difficulty	2 (33.3)	3 (50.0)	1 (16.7)	
Walking				0.041
No difficulty	0	5 (45.5)	6 (54.5)	
At least some difficulty	12 (31.6)	14 (36.8)	12 (31.6)	
Self-care^2^				0.002
No difficulty	0	6 (37.5)	10 (62.5)	
At least some difficulty	11 (34.4)	13 (40.6)	8 (25.0)	
Communication				0.018
No difficulty	5 (16.7)	10 (33.3)	15 (50.0)	
At least some difficulty	7 (36.8)	9 (47.4)	3 (15.8)	
Learning				0.186
No difficulty	4 (20.0)	6 (30.0)	10 (50.0)	
At least some difficulty	8 (27.6)	13 (44.8)	8 (27.6)	
Remembering				0.282
No difficulty	5 (20.0)	9 (36.0)	11 (44.0)	
At least some difficulty	7 (29.2)	10 (41.7)	7 (29.2)	
Controlling behaviour				0.018
No difficulty	5 (16.7)	10 (33.3)	15 (50.0)	
At least some difficulty	7 (36.8)	9 (47.4)	3 (15.8)	
Completing a task^2^				0.114
No difficulty	5 (19.2)	8 (30.8)	13 (50.0)	
At least some difficulty	6 (27.3)	11 (50.0)	5 (22.7)	
Accepting change				0.763
No difficulty	9 (29.0)	10 (32.3)	12 (38.7)	
At least some difficulty	3 (16.7)	9 (50.0)	6 (33.3)	
Getting along with other children				0.005
No difficulty	4 (14.3)	9 (32.1)	15 (53.6)	
At least some difficulty	8 (38.1)	10 (47.6)	3 (14.3)	
Feeling worried/sad^3^				0.874
Never experienced	8 (28.6)	9 (32.1)	11 (39.3)	
At least a few times a year	4 (19.0)	10 (47.6)	7 (33.3)	

In addition to walking, the level of schooling was also significantly associated with the ability for self-care (p<0.001), communication (p<0.002), controlling behaviour (p=0.008) and getting along with other children (p<0.001) (Table 4).

Barriers and facilitators to education

The five themes underpinning the parents and caregivers' perception of barriers and facilitators to education were ‘school infrastructure’, ‘inclusive opportunity, staffing and teacher’s training’, ‘social environment’, ‘child’s intrinsic factor’ and ‘practical limitation’.

School Infrastructure

Out of 46 CWND who were attending school, unsuitable school physical structures and facilities were among the most reported barriers (n=17, 36%). Within this group, almost all CWND were rated as at least ‘some difficulties’. Difficulties in accessing the classroom, toilet, canteen and library were mentioned. Many of the children in this study had classrooms or libraries situated above the ground floor, and there were no lifts available. Some toilets in schools were too far, the door was too narrow for wheelchair entry, steps at the entrance or simply unsuitable (squat type rather than the sitting type, which was inconvenient for children with difficulty squatting down). A few parents commented that there was no ramp for wheelchair access in school areas. One caregiver reported that the queuing lane in the canteen was not wide enough to accommodate a wheelchair, and therefore, the child could not buy things at the canteen counter. Most of the CWNDs in wheelchairs relied on teachers or peers to move them around the school.

“The library is on the second floor and he couldn’t go there because he couldn’t climb the stairs.” (P41, congenital muscular dystrophy, ‘a lot of difficulty’ in mobility domain.)

“His class is on the third floor and he will have to climb the stairs. He can go up the stairs himself, but there is risk of falling.” (P31, inborn error of metabolism, ‘some difficulty’ in mobility domain.)

“Her classroom is on the first floor…She eats her packed food in the class alone during recess.” (P51, spastic diplegia, ‘cannot do’ in mobility domain.)

One child in this study was denied entry into mainstream class because the classes were not on the ground floor.

“His academic results are good, but he can’t enter mainstream class because the class is not on the ground floor. He is very disappointed. I have visited several schools to meet the school principal and could not get him into mainstream class. There are no schools with lifts.” (P5, spinal muscular atrophy type 3, ‘cannot do’ in the mobility domain.)

Reasons related to the physical environment of the school were also reported by two of the caregivers of CWND for not attending school.

“The school is unsuitable [for him]. It is very difficult to carry him into the class. There is no facility in the school for changing diapers.” (P40, Duchenne muscular dystrophy, ‘cannot do’ in the mobility domain.)

“The school is on top of the hill and there is no special lane in the school compound for wheelchair.” (P42, Duchenne muscular dystrophy, ‘cannot do’ in the mobility domain.)

Two caregivers suggested having classrooms on the ground floor as one of the facilitators of education. One caregiver whose child is in mainstream education shared that the school had decided to keep his child on the ground floor throughout primary 1 to 6 to accommodate the child’s mobility difficulties.

Inclusive Opportunity, Staffing and Teacher Training

Twelve caregivers of CWND (26%) in schools stated that their children were not participating in sporting activities while they were in school. Within this group, three caregivers described that their child sat at the side and watched others play during physical education lessons, and one caregiver stated that the child was exempted from sporting activities by the teacher. Around half of these children (n=7, 58%) in this group were rated as having 'a lot of difficulty' or ‘cannot do’ when asked if they are able to walk (aided or unaided) 100 metres on level ground and the rest of the 41% were rated as 'some difficulties' in the same category. One caregiver stated that the child was not allowed to join class outing activities without accompanying caregivers due to staff shortage. One barrier stated by four caregivers was the placement of CWND with diverse needs in the same class and one caregiver described the lack of knowledge and training in dealing with children with varying abilities among the teachers.

“There is no separate class for children with cerebral palsy. Children with different diagnoses such as autism and Down syndrome who can write independently are in the same class [as her]. Teachers asked me to accompany her in school as she can’t write or join activities otherwise. I have raised this issue with the school committee but they told me that they need 5 new children to justify starting a separate class." (P28, Spastic Quadriplegic Cerebral Palsy with bilateral hip dislocation, GMFCS V, ‘cannot do’ in the mobility domain.)

“The school has equipment such as standing frame, ball and corner chair. But teachers are not trained to use them. I am the one who use it for my child instead.” (Spastic Quadriplegic Cerebral Palsy with bilateral hip dislocation, GMFCS V, ‘cannot do’ in the mobility domain.)

Three caregivers of CWND stated that their child needed help from peers to go to the toilet as one of the barriers faced in school. Among 16 CWND who were not in school, five caregivers (31%) explained the reason was a lack of assistance with self-care.

“Since his operation 12 months ago, he has difficulty gripping with his left hand, if he needs to go the toilet, nobody can help him (in school).” (P18, brain tumour with hemiparesis, ‘some difficulty’ in the mobility and self-care domain.)

“He is not using diapers at home and his grandmother usually carries him to the toilet. No one can help him with that in school.” (P42, Duchenne muscular dystrophy, ‘cannot do’ in the mobility domain and ‘some difficulty’ in the self-care domain.)

“She can’t care for herself, talk or communicate with others.” (P19, drug-resistant epilepsy, ‘cannot do’ in the mobility, self-care and communication domain.)

“We are required to be with the child throughout the session in school as my child is unable to care for himself. We do not have the time.” (P20, Spastic dystonic cerebral palsy, GMFCS V, ‘cannot do’ in the mobility, self-care and communication domains.)

One parent decided against sending her child to school after advice from the school authority.

“He completed mainstream primary school education. But after one week of starting his secondary school, we were called in by the school principal and told that he should go to PPKI (integrated special education program) because he might get bullied by others in the [mainstream] school. [The principal] said the school is located on top of a hill and not suitable for the child due to his disability.” (P55, Duchenne muscular dystrophy, ‘cannot do’ in the mobility domain.)

Among 46 CWND in school, the most common facilitators of education mentioned among the caregivers were improving the quality and quantity of teachers and modifying the teaching curriculum. More than a third of them (n=17, 37%) mentioned the need to increase the staffing including having more teachers and teacher assistance to help improve the student:teacher ratio in class to meet their child’s needs in class. Two caregivers in this group expressed their wish of having 1:1 support in class. Five caregivers highlighted the need for teacher training.

“Teachers have to focus on the difficulties of the child and try to develop skills according to each individual needs.” (P1, Spastic Diplegic Cerebral Palsy, GMFCS II, ‘a lot of difficulty’ in mobility, learning and memory domain and ‘some difficulty’ in communication domain.)

There were nine caregivers who felt that there should be a change in class scheduling and teaching method. This includes two caregivers who mentioned the need for reasonable adjustment in school such as allowing more time between lessons so that the child could move from one location to another and allowing children to leave for recess at the canteen five minutes earlier. Another caregiver suggested organising more outdoor activities such as horse riding.

Social Environment

Two caregivers described school bullying as a barrier to education. Four caregivers (25%) out of 16 caregivers who did not send their child to school said that they were afraid that the child may be hurt in school by other children in school.

“I went [to school] with her once. Other students disturb her. They put food in her mouth and touch her face. I feel uneasy to send her to school as children with diverse needs are together in the same class.” (P62, spastic quadriplegic cerebral palsy, GMFCS V, ‘cannot do’ in mobility, self-care and communication domain.)

“He stopped schooling since his brain operation. His legs are weak and other children might push him. I don’t feel safe. There are 40 students to 1 teacher.” (P39, brain tumour, ‘some difficulty’ in mobility and self-care domain and ‘no difficulty’ in the other domains.)

“I am worried that he would get bullied in school by other children.” (P55, Duchenne Muscular Dystrophy, ‘cannot do’ in the mobility domain, ‘some difficulty’ in self-care domain and ‘no difficulty’ in the other domain.)

“I am worried that other children will disturb him. Others (teachers) do not know his needs.” (P36, spastic quadriplegic cerebral palsy, GMFCS V, ‘cannot do’ in the self-care mobility domain and ‘a lot of difficulty’ in communication domain.)

Child Intrinsic Factors

Thirty-two (51%) and 38 (61%) caregivers in the study rated that their CWND had at least 'some difficulties' in learning and memory, respectively, when completing the Washington group/UNICEF Child Functioning Module’ (CFM). Sixteen (35%) caregivers among 46 CWND who were attending school stated learning issues as one of the barriers. These include difficulties in reading, copying, writing, counting, remembering and understanding instructions. Around half of the caregivers (n=30, 48%) rated at least 'some difficulties' in attention yet only seven mentioned attention as the barrier in their open-ended questions responses. Five caregivers reported that the main barriers that their child faced were difficulties either in communication or making friends in school.

Practical Limitations

This was among the most salient themes reported by caregivers when asked about the reason(s) their child was out of school in this study. Reasons provided include long distance to school, lack of transport, financial difficulty, time constraints and having other younger children at home that caregivers needed to look after.

“The school is far away from home. I don’t have any transport to send him to school and I have a younger child at home to look after.” (P47, inborn error of metabolism, ‘cannot do’ in the mobility, self-care and communication domains.)

“The school transport driver … lacks strength to carry him into the vehicle. So, he has to stop schooling since he stopped ambulating.” (P42, Duchenne muscular dystrophy, ‘cannot do’ in the mobility domain, ‘some difficulty’ in self-care domain and ‘no difficulty’ in communication domain.)

“We have no time to bring her to school. Both of us (parents) need to work due to financial difficulties.” (P60, Lissencephaly, ‘cannot do’ in the mobility, self-care and communication domain.)

## Discussion

To the best of our knowledge, this is the first study to explore and discuss the parental perception of the barriers and facilitators to education among CWND in Malaysia. Less than 40% of the CWND were in mainstream schools, and over 25% were not in school. There was an association between the level of schooling and the ability to walk, self-care, communication, controlling behaviour and getting along with other children. Our study also highlighted the five themes underpinning these barriers and facilitators to education: ‘school infrastructure’, ‘inclusive opportunity, staffing and teacher’s training’, ‘social environment’, ‘child’s intrinsic factor’ and ‘practical limitation’.

In Malaysia, it was estimated that overall, around 4.5% of children do not attend school [16]. In our study, the rate of children aged seven or more being out of school was 24%, i.e. five times more than the estimated national figure of 4.5%. Our figure is comparable to the prevalence rate of 31% among children with physical disability reported in a Malaysia population-based study [5]. The out-of-school rate in our study is lower than the rate reported in a UNICEF study among children with disability in Sabah (50% to 69%) which is the poorest state in Malaysia [12]. The difference may be related to a relatively small sample size, and this study was conducted in a tertiary hospital attended by people from the urban region.

School infrastructure including accessibility to classrooms, toilets, canteen and library was the most frequently reported theme in this study. Many schools that the CWND attended in this study appeared to have facilities that were less wheelchair friendly. A popular Malaysian news portal recently reported the story of an eight-year-old girl with cerebral palsy who was a wheelchair user being carried by her mother onto the first floor of the school building since she entered school two years prior [17]. According to the news report, the school authority had initially suggested that the girl be placed in a special education class which was situated on the ground floor, but the girl did not have a learning disability. Sadly, there were no lifts or ramps available in that school. Stories like these are common and echo what we found in our study. The children aged seven or more in our study who were out of school were also more likely to be wheelchair users than those in school, and the level of schooling was strongly associated with the ability to walk. A previous study exploring how different types of disabilities can affect school attendance and completion in eight developing countries reported that primary schools appeared to be less accommodating for children with mobility difficulties than other types of disabilities [18]. It was indeed encouraging to hear from one caregiver in this study who shared that the school authority had made adjustments for the child such that the classroom remained on the ground floor from primary 1 to primary 6, even though typically students are supposed to move into a different classroom which may be situated at different levels of the building within the school. However, reasonable adjustment like this seems to be an exception rather than the norm. The Malaysian Ministry of Education has recognised that one of the barriers faced by students with disability is less accommodating infrastructure and the lack of important features such as ramps, lifts or disabled toilets [19]. The lack of school facilities to accommodate children with disability is also highlighted in a qualitative study done among special education teachers in Malaysia [20]. This finding was supported by another study which reported the lack of facilities, resources and teaching materials [21]. Overall, the unmet educational need of CWND in terms of school infrastructure remains stark.

Children with disability are more prone to reduced school participation [22]. School participation means being involved in activities inside and outside the classroom (such as clubs, sports, art, group work and study) as well as activities during recess such as playing at the school playground and hanging out with friends [23]. In a qualitative study done in Malaysia, the need for outdoor activities was highly expressed by parents of children with severe cerebral palsy and the low participation level of children with cerebral palsy was suggested [20]. Similarly, in our study, participation in sports activities was a relatively common issue raised by caregivers of CWND attending school. Among parents who raised this issue, 58% of these children had significant mobility difficulties and it was surprising that a considerable proportion (41%) were rated as only having 'some difficulties' when asked if the child can walk 100 metres on level ground. One may expect substantially better participation levels if they have only 'some difficulties' in mobility. This may be related to the lack of an inclusive environment (for example, the lack of suitable infrastructure, sporting activities and inadequate training of the educators, which are the other themes emerging from this study). This trend was similar to the finding of a large New Zealand household disability survey which found that a surprisingly high proportion of children with physical disability (59%) were prevented from joining sports in school despite the sample of children in the study had a varying level of physical abilities and could range from having mild handwriting problems to mobilising in a wheelchair [24]. In addition to reduced inclusive opportunities for sport activities, caregivers in this study also reported reduced participation in academic activities, social activities and usage of learning aid compared to their peers.

Overall, 17.5% of all the caregivers whose children are in school in this study reported that their child was bullied. In the open-ended responses, several caregivers identified bullying as a barrier to education. The fear of CWND being bullied in school was also a reason why some caregivers did not send their child to school in our study. A UNESCO report in 2021 summarising data from various countries stated that students with disabilities are more likely to be victims of school bullying than their peers at all ages and in all educational settings; this negatively impacts their education, health and well-being [25]. A UNICEF survey on knowledge, attitude and perception regarding children with disability in Malaysia highlighted the vulnerability of these children to bullying and mistreatment which can include name-calling, teasing, mocking or physical beatings [26]. The UNICEF study reported that children with disability are often bullied, and the study participants who include adolescents with disability expressed that the society and the existing services did not protect them sufficiently from bullying and abuse [26].

Children in our study with no difficulty in self-care, communication, controlling behaviour and getting along with other children are more likely to be in mainstream school. A potential explanation is that CWND who have difficulties in these domains may require more support from the school teachers who will have to teach in a class with an average classroom size of 29 students (Malaysian Education blueprint). Among 46 children who were in school in our study, 25 (56%) were in mainstream education and 16 (35%) were in the Integrated Special Education Program (also known as 'PPKI'). 'PPKI' is a programme that is carried out in specific classes within mainstream school facilities for children with special needs and the guidelines from the Ministry of Education stipulate that the student-teacher ratio should be less than 7:1 [27]. The ‘PPKI' class is situated on the ground floor, and children are divided into different classes within the programme based on their educational needs. In one Malaysia-based study, there was a great need for special schools for children with severe cerebral palsy and the author suggested that this may be due to the limited availability of special education programme among public schools in Malaysia [20]. In 2020, there were 2,472 primary and secondary schools in Malaysia with the Integrated Special Education Program attended by around 74,000 students [28]. In our study, practical limitations such as distance to school and transport difficulty were among the most common reasons that some CWND were not in school. Perhaps increasing the availability of schools with Integrated Special Education Program may help to improve the high out-of-school rate among CWND.

In developed countries such as the United Kingdom, there are school-based therapists such as speech therapists or occupational therapists for children with special education needs in school settings. They also provide class support to the teacher and advice to caregivers. Every school in the UK is also required by law to have a Special Educational Needs Coordinator (SENCo) who are members of the teaching staff tasked to ensure that the needs of students with special education needs are met and that appropriate adjustments are made within the school [29]. In Malaysia, the Ministry of Education has set up regional centres called the Special Education Service Centre also known as '3PK' aiming to provide rehabilitation, interventional and consultation services in the areas of audiology, psychology, speech therapy and occupational therapy. The centres are staffed by teachers who furthered their studies in the relevant field, and they can work with parents and caregivers to explore ways and strategies to meet a child’s educational needs in the school setting. However, there are only 13 such centres in Malaysia and this service does not reach many regions within Malaysia [27]. With that, healthcare professionals who are reviewing CWND in hospitals can play important roles in improving the children’s educational experience. First, the healthcare professionals can provide a comprehensive multidisciplinary assessment of individual functional levels and needs, taking into account the school environmental factors which are in play. Then, the findings of the assessment can be communicated to the school to facilitate the school authority in making reasonable adjustments for the child. Part of the assessment can also involve a school visit by the healthcare team to enable the prescription and usage of suitable adaptive devices in school and provide suggestions for required facilities. This two-pronged approach consisting of individual and environmental intervention can help to enhance school participation among children with disability [30]. Improving teachers’ knowledge is also essential to enhance school participation among children with disability [30]. Training held by healthcare professionals on specific topics such as sporting activities suitable for children with disability, augmentative and alternative communication, behavioural management, teaching emotional regulation or acute seizure management can empower the teachers to provide better support to CWND in class. Lastly, healthcare professionals can also be the point of contact for the setup of parental support groups or parent-based associations whose voices can later become instrumental in the advocacy of the educational rights of their children. This may potentially become a push factor for improvement within the educational system to better support CWND.

Our study has several limitations. Firstly, the sample size is relatively small and the sampling was not random. Most of the CWND in the study had cerebral palsy, and those with visual/hearing difficulties were under-represented. The study only captured information from CWND who attended appointments at a government tertiary hospital in an urban area and may not be representative of CWND from rural regions or those who engaged less well with hospital follow-ups. Future studies should be done with a larger sample size involving parents from rural regions. This study investigates parental perception of the child’s functioning level and parents may overrate or underestimate their child’s difficulties. Children’s personal perspectives were not explored in this study; this is a potential point for research in the future.

## Conclusions

This study highlighted the significant educational barriers that CWND are facing, especially less accommodating school infrastructure and staffing issues. There is a dire need for practical solutions to overcome these barriers, for example, increasing the availability of schools with Integrated Special Education Program. This study also found that the level of schooling was strongly associated with difficulties in mobility, self-care, communication, controlling behaviour and getting along with other children. Findings from this study can be helpful for healthcare professionals, educators and policymakers to improve the accessibility and experience of education among CWND, which may then assist each child in achieving their full learning and developmental potential.
